# Reply to ‘Screening for cardiac sarcoidosis: diagnostic approach and long-term follow-up in a tertiary centre’

**DOI:** 10.1007/s12471-025-01995-8

**Published:** 2025-10-06

**Authors:** Nikki van der Velde, Alexander Hirsch

**Affiliations:** Department of Cardiology, Radiology and Nuclear Medicine, Erasmus MC, Rotterdam, The Netherlands

We thank Drs. de Leeuw and colleagues for their interest and thorough review [1] of our published manuscript on screening for cardiac sarcoidosis [2]. The authors have several questions regarding the best screening algorithm for patients with extracardiac sarcoidosis. Cardiac MRI and/or fluorodeoxyglucose positron emission tomography (PET) can detect cardiac sarcoidosis more accurately than standard transthoracic echocardiography (TTE); however, these methods are costly, require specialized expertise for interpretation, involve radiation exposure in the case of PET, and are unlikely to be practical for routine use in all individuals with sarcoidosis [3]. We agree that speckle-tracking TTE appears promising for detecting cardiac sarcoidosis [3, 4]; unfortunately, this was not available in our cohort.

As depicted in our study, a normal ECG and TTE do not fully exclude the presence of cardiac sarcoidosis; however, it was reassuring that the prognosis for these patients is good. Nonetheless, based solely on this observation, we cannot conclude that withholding treatment from these patients is justified.

Despite the clear limitations, we still recommend further testing based on symptomatology and ECG as the initial screening tool [3]. However, there should be a low threshold to order advanced cardiac imaging in patients with established extracardiac sarcoidosis [5].
